# Fishing-Injury-Related Flexor Tenosynovitis of the Hand: A Case Report and Review

**DOI:** 10.1155/2013/587176

**Published:** 2013-01-15

**Authors:** Danny A. Young-Afat, Deniz Dayicioglu, John C. Oeltjen, Audene P. Garrison

**Affiliations:** ^1^University of Amsterdam, The Netherlands; ^2^Division of Plastic and Reconstructive Surgery, Department of Surgery, University of Miami Miller School of Medicine, USA; ^3^Division of Plastic Surgery, Department of Surgery, University of South Florida, USA; ^4^Division of Infectious Diseases, University of Miami Miller School of Medicine, USA

## Abstract

Hand infections occurring after fishing and other marine-related activities may involve uncommon bacteria that are not susceptible to the conventional or empiric antibiotic therapy used to treat soft tissue infections. Therefore appropriate treatment is often delayed and could lead to severe hand damage. An illustrative case of fishing-related injury leading to complicated tenosynovitis and horseshoe abscess caused by *Mycobacterium marinum* and its treatment course is outlined. Laceration of the skin during boating is fairly common. Because of the rarity of some of the bacteria, referrals to the appropriate specialist including hand surgeons and infectious disease specialists should occur in early stages. *M. marinum* infections should always be considered in injuries related to seawater and fishing as this may lead to early appropriate treatment and prevent severe damage.

## 1. Introduction

An otherwise healthy 65-year-old right-hand-dominant man was referred with numbness in the median nerve distribution and tenosynovitis of the left small finger and thumb.

**Figure 1:**
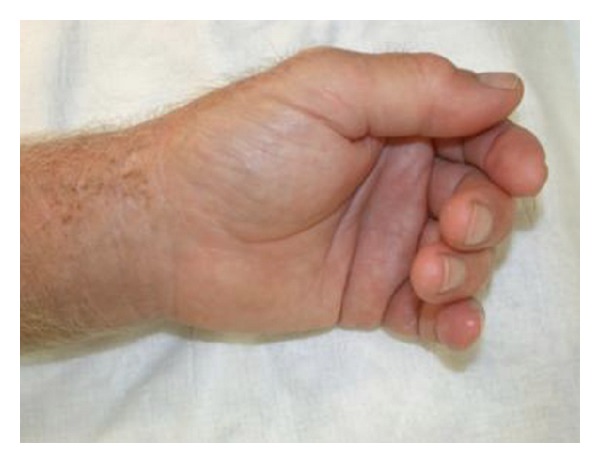
Swelling and erythema on the volar aspect of the small finger and thumb of the left hand.

**Figure 2:**
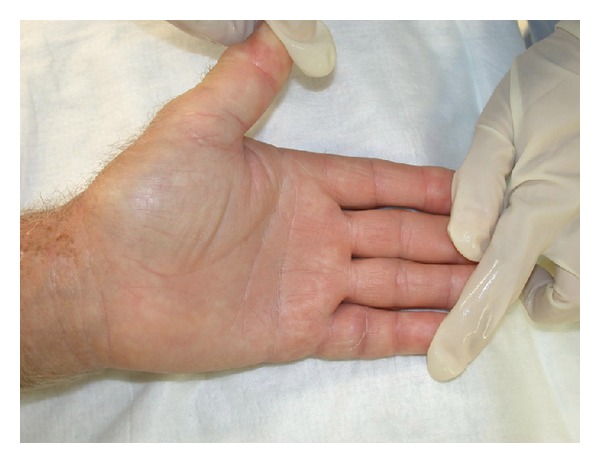
Swelling and erythema on the volar aspect of the small finger and thumb of the left hand.

**Figure 3:**
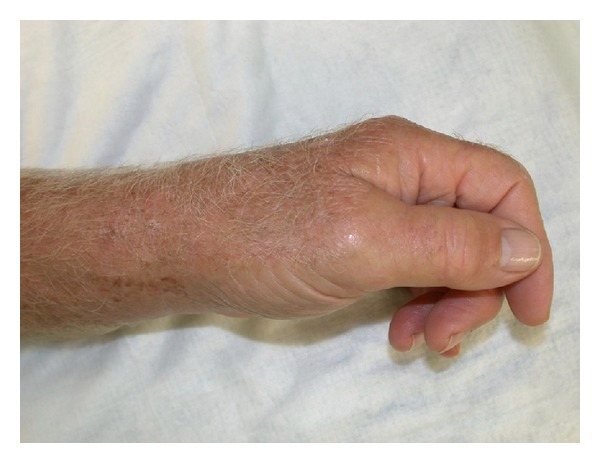
Swelling and erythema on the dorsal aspect of the small finger and thumb of the left hand.

The patient recalled a penetrating injury with a barnacle in the flexor crease of his nondominant left-hand small finger 2 months prior to presentation. The initial laceration healed without any problems. The erythema persisted and the swelling did not resolve, limiting the range of motion in the small finger at the proximal interphalangeal joint level. He was concerned about a foreign body and went to an emergency room 1 month prior to presentation at our hand surgery service. X-rays at that time demonstrated no evidence of phalangeal fracture, bony involvement, or presence of a foreign body. He was treated empirically with two courses of amoxicillin/clavulanic acid for ten days. His symptoms failed to resolve on this regime and his antibiotic therapy was changed to doxycycline for ten days. He continued to experience swelling and erythema involving the small finger. Two months after the initial injury, the patient presented to the ER again. This time with swelling and erythema involving the small finger and the thumb and a painful wrist (Figures 1, 2, and 3).

 The pulses were palpable; the capillary refill was within normal ranges. His wrist and thumb were also swollen and painful on passive and active ranges of motion. He had no fever and his white blood cell count was 7.3 K/cmm. The magnetic resonance imaging taken in the emergency department demonstrated inflammation extending around the small finger flexor tendon proximally to the region of the carpal tunnel and also to the volar aspect of the hand and into the thumb. The findings were reminiscent of a horseshoe abscess of the hand. The patient was then admitted, and the horseshoe abscess with associated tenosynovitis was treated with surgical drainage under general anesthesia.

**Figure 4:**
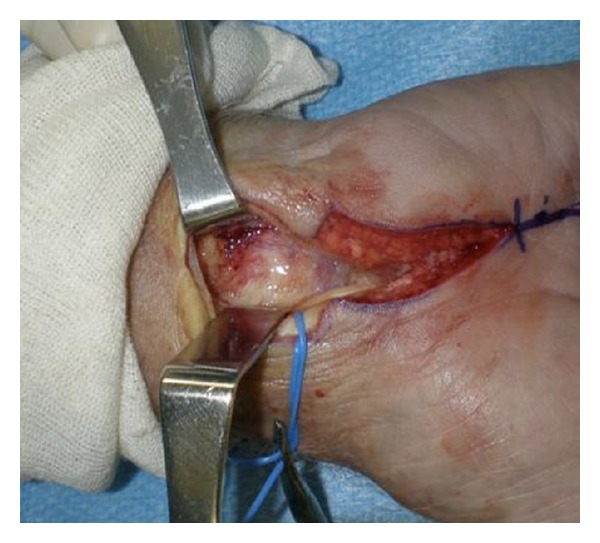
The left hand with marked fluid and proliferation of the synovial sheaths in the carpal tunnel.

**Figure 5:**
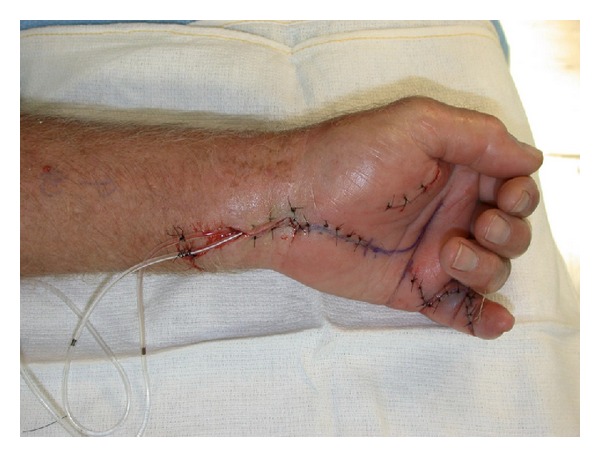
The left hand with irrigation system consisting of a pediatric feeding tube size number 5 Fr inserted into the synovial sheath with open-tube drainage. Loosely sutured skin to allow drainage.

 Brunner incisions were used to drain the small finger; an oblique incision was made for thenar and hypothenar spaces and a carpal tunnel release incision was done. No evidence of pus was revealed; however, a marked clear fluid was evident throughout the hand. There was marked proliferation of synovial sheaths in the carpal tunnel. Cultures for aerobic, anaerobic, acid fast bacilli, and fungus were taken along with a synovial sheath biopsy (Figure 4). The wound was copiously irrigated with saline and an irrigation system consisting of a pediatric feeding tube size number 5 Fr was inserted into the synovial sheath with open-tube drainage. The skin was loosely sutured to allow drainage (Figure 5). The Infectious Diseases Service was consulted and an empiric therapy was commenced with vancomycin, piperacillin/tazobactam, ethambutol, and Rifampin. *M. marinum* was strongly suspected due to the chronicity of the hand infection and the history of injury with seawater exposure.

 Significant improvement in the inflammation was observed after surgery. Erythema and swelling in the wound decreased and the irrigation system was removed on the 3rd day. At the same day, active and passive ranges of motion exercises were started, and the patient demonstrated increased mobility and good response to therapy throughout treatment.

**Figure 6:**
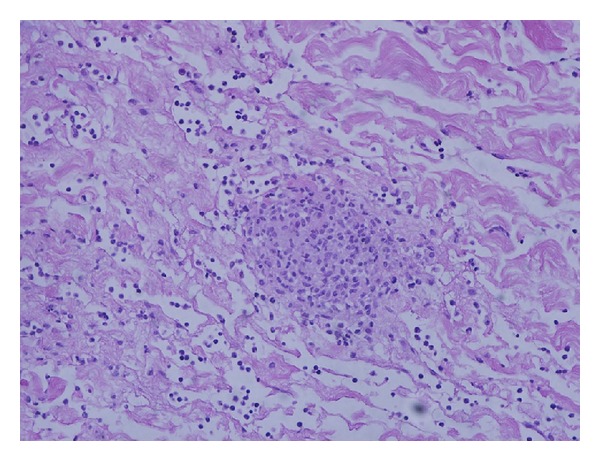
Pathology specimen demonstrating granulomatous tenosynovitis with acid fast bacilli.

**Figure 7:**
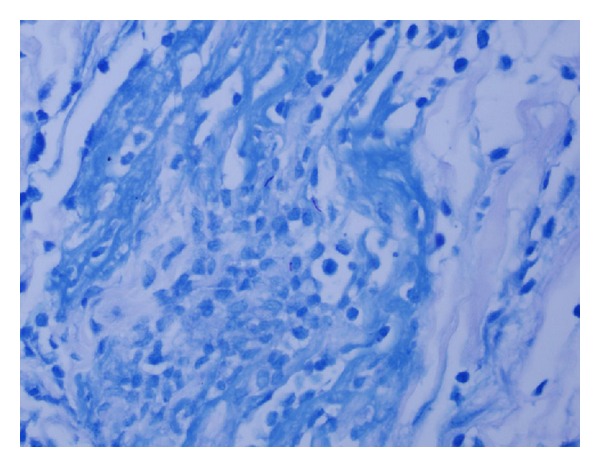
Mycobacterial tissue cultures demonstrating *Mycobacterium marinum*.

The bacterial smear and culture did not demonstrate or isolate any bacterial pathogen, respectively. The pathology specimen demonstrated granulomatous tenosynovitis with acid fast bacilli (Figure 6). Two and a half weeks after his surgical procedure, *M. marinum* was isolated from mycobacterial tissue cultures (Figure 7). His antibiotic regime was subsequently changed to clarithromycin 500 mg twice daily, as he was adamant about minimizing his medication burden. He continued with clarithromycin as an outpatient. His horseshoe abscess and tenosynovitis healed without evidence of recurrence after 3 consecutive months.

## 2. Discussion

 The course of the disease is insidious in *M. marinum* tenosynovitis as it is often misdiagnosed upon initial presentation. Most patients present with atypical symptoms including pain, swelling, and lack of movement. In later stages, tendon thinning and tendon rupture can occur.

 Horseshoe abscess is a complication of a flexor tenosynovitis occurring after progression of the infection following the synovial communication between the radial and ulnar bursae at the wrist level. As is seen in this case, involvement of the small finger tendon sheath extends to the ulnar bursa. The ulnar bursa communicates with the radial bursa in the wrist level at the potential Parona's space. By this route, the infection extends to the thumb flexor sheath. The horseshoe abscess can be drained by a combination of small finger-ulnar bursae and flexor pollicis radial bursa incisions [1]. The patient also had decreased sensation in the median nerve distribution. Pus in the thumb flexor sheath can also lead to infection in carpal tunnel causing the carpal tunnel syndrome symptoms [1–3].

*M. marinum* is a nontuberculous mycobacteria found in aquatic environments, fresh and seawater, fish tanks, and nonchlorinated swimming pools. Among the vectors of infection are marine organisms such as fresh or saltwater fish and shellfish. The estimated incidence of *M. marinum* soft tissue infection is 0.27 per 100,000 [4]. It typically causes a chronic granulomatous soft tissue infection. The incubation period ranges from 2 to 6 weeks. After trauma and contact with *Mycobacterium marinum*, usually a solitary papule appears on the extremity enlarging to a nodule or plaque progressing at times to ulceration—“Swimming pool granuloma.”

 Rarely involvement of the proximal lymph nodes may progress to scrofuloderma [4, 5].

 Physician's awareness of *Mycobacterium marinum* is necessary to treat this slow infection early [6]. Delay in treatment can cause direct extension of the cutaneous infection to the deep structures causing infiltration of the tendon sheath, bursae, joints, and bones in 29% of reported *M. marinum* infections [7]. Tenosynovitis is the most common infection involving deeper tissues [5]. The disseminated disease is rare and occurs in immunosuppressive therapy as in transplant recipients and rheumatoid patients [8, 9].

 The diagnosis is based on the clinicohistological features and response to treatment [6]. Diagnosis involves both biopsy of the involved tissue for histopathological evaluation and culture. Synovial lesions may resemble a rheumatoid disease. The treatment is debridement and pharmacological therapy [1]. After debridement, large defects can be left open or vacuum-assisted closure could be used [10]. In our patient, although there was no pus, synovial tissue proliferation and clear fluid was evident through the small finger and flexor sheath and flexor pollicis longus tendon sheath involving the tendons in the carpal tunnel. Although the disease is indolent, rapid progression of the disease leading to index finger amputation is also reported [11]. Deep infections can cause significant damage with marked proliferation if in the synovial tissue, erosion of the joints, involvement of the flexor and extensor tendons. Pus may be evident tracking into the forearm or to the potential spaces in hand [12].

*M. marinum* may be isolated from cultures in two to three weeks. This mycobacterium species grows optimally at lower temperatures of between 28 and 33°C. It is, therefore, important to notify the microbiology laboratory of the possibility of this species so that cultures are incubated at the appropriate temperature.

 Susceptibility testing of *M. marinum* isolates demonstrates susceptibility to rifampin, rifabutin, ethambutol, clarithromycin and sulfonamides, and trimethoprim/sulfamethoxazole. It may demonstrate full or intermediate susceptibility to doxycycline and minocycline and intermediate susceptibility to streptomycin. It is resistant to isoniazid and pyrazinamide [13, 14].

 The natural course for *M. marinum* infections is slow spontaneous resolution in 1–6 years. The aim of the treatment of superficial lesions is to prevent the rare progression to deep infection [12]. The localized or superficial disease may be treated with a single agent by some specialists.

 Good outcomes have been reported for combinations with clarithromycin and ethambutol or ethambutol and rifampin. A rifampin-based regime is recommended for cases with osteomyelitis [14]. There have been no published randomized controlled trials comparing the various treatment regimes for soft tissue infection caused by *M. marinum*. For superficial infections, the antibiotic therapy is given for 6 weeks to 6 months. The optimal treatment period is determined by clinical response [9]. For deep infections, the duration of the therapy is for 6 to 18 months [12]. The patient should be controlled for reactivation of the disease after the discontinuation of the medication. The American Thoracic Society/Infectious Disease Society of America recommends treatment with two active agents for one to two months after resolution of lesions, approximately three to four months of total therapy [14].

 Although rare, hand infections caused by injuries related to fishing can result in serious complications. Waterborne infections can be acutely fulminant, such as necrotizing fasciitis that results from “*Vibrio*” infections or chronic in nature that include atypical “mycobacteria.” Chronic infections are often underdiagnosed and there is often a delay in treatment. *M. marinum* infection should be considered early when patients present with a fishing-related injury such as a cut with a hook, exposure to fish, and small lacerations with shells that are problematic to heal. Early recognition and treatment of fishing-related hand infections could prevent severe damage to vital structures in the hand.
